# Supplementary material for: Modelling Skylarks (Alauda arvensis) to Predict Impacts of Changes in Land Management and Policy: Development and Testing of an Agent-Based Model
Source: PLoS One. 2013 Jun 6;8(6):e65803. doi: 10.1371/journal.pone.0065803 (PMC3675089; doi:10.1371/journal.pone.0065803)
Supplement: Supporting Information S4 — The skylark ODdox as a zipped archive. (ZIP) [file pone.0065803.s004.zip › Skylark_ODdox/treatment_8h.html]

ALMaSS Skylark ODdox: treatment.h File Reference


ALMaSS Skylark ODdox
 2.0


- Main Page
- Related Pages
- Classes
- Files

- File List
- File Members

Enumerations

treatment.h File Reference

|  |  |
| --- | --- |
| Enumerations | |
| enum | FarmToDo {     start = 1, sleep\_all\_day, autumn\_plough, autumn\_harrow,     autumn\_roll, autumn\_sow, winter\_plough, deep\_ploughing,     spring\_plough, spring\_harrow, spring\_roll, spring\_sow,     fp\_npks, fp\_npk, fp\_pk, fp\_liquidNH3,     fp\_slurry, fp\_manganesesulphate, fp\_manure, fp\_greenmanure,     fp\_sludge, fa\_npk, fa\_pk, fa\_slurry,     fa\_ammoniumsulphate, fa\_manure, fa\_greenmanure, fa\_sludge,     herbicide\_treat, growth\_regulator, fungicide\_treat, insecticide\_treat,     molluscicide, row\_cultivation, strigling, flammebehandling,     hilling\_up, water, swathing, harvest,     cattle\_out, pigs\_out, cut\_to\_hay, cut\_to\_silage,     straw\_chopping, hay\_turning, hay\_bailing, stubble\_harrowing,     autumn\_or\_spring\_plough, burn\_straw\_stubble, mow, cut\_weeds,     strigling\_sow, trial\_insecticidetreat, trial\_toxiccontrol, trial\_control,     syninsecticide\_treat, cattle\_out\_low, product\_treat, glyphosate,     last\_treatment   } |

---

## Enumeration Type Documentation

enum FarmToDo

**Enumerator:**
:   |  |  |
    | --- | --- |
    | *start* |  |
    | *sleep\_all\_day* |  |
    | *autumn\_plough* |  |
    | *autumn\_harrow* |  |
    | *autumn\_roll* |  |
    | *autumn\_sow* |  |
    | *winter\_plough* |  |
    | *deep\_ploughing* |  |
    | *spring\_plough* |  |
    | *spring\_harrow* |  |
    | *spring\_roll* |  |
    | *spring\_sow* |  |
    | *fp\_npks* |  |
    | *fp\_npk* |  |
    | *fp\_pk* |  |
    | *fp\_liquidNH3* |  |
    | *fp\_slurry* |  |
    | *fp\_manganesesulphate* |  |
    | *fp\_manure* |  |
    | *fp\_greenmanure* |  |
    | *fp\_sludge* |  |
    | *fa\_npk* |  |
    | *fa\_pk* |  |
    | *fa\_slurry* |  |
    | *fa\_ammoniumsulphate* |  |
    | *fa\_manure* |  |
    | *fa\_greenmanure* |  |
    | *fa\_sludge* |  |
    | *herbicide\_treat* |  |
    | *growth\_regulator* |  |
    | *fungicide\_treat* |  |
    | *insecticide\_treat* |  |
    | *molluscicide* |  |
    | *row\_cultivation* |  |
    | *strigling* |  |
    | *flammebehandling* |  |
    | *hilling\_up* |  |
    | *water* |  |
    | *swathing* |  |
    | *harvest* |  |
    | *cattle\_out* |  |
    | *pigs\_out* |  |
    | *cut\_to\_hay* |  |
    | *cut\_to\_silage* |  |
    | *straw\_chopping* |  |
    | *hay\_turning* |  |
    | *hay\_bailing* |  |
    | *stubble\_harrowing* |  |
    | *autumn\_or\_spring\_plough* |  |
    | *burn\_straw\_stubble* |  |
    | *mow* |  |
    | *cut\_weeds* |  |
    | *strigling\_sow* |  |
    | *trial\_insecticidetreat* |  |
    | *trial\_toxiccontrol* |  |
    | *trial\_control* |  |
    | *syninsecticide\_treat* |  |
    | *cattle\_out\_low* |  |
    | *product\_treat* |  |
    | *glyphosate* |  |
    | *last\_treatment* |  |

{

start = 1, // 1, must be first.

sleep\_all\_day,

autumn\_plough,

autumn\_harrow,

autumn\_roll,

autumn\_sow,

winter\_plough,

deep\_ploughing,

spring\_plough,

spring\_harrow, // 10

spring\_roll,

spring\_sow,

fp\_npks,

fp\_npk,

fp\_pk,

fp\_liquidNH3,

fp\_slurry,

fp\_manganesesulphate,

fp\_manure,

fp\_greenmanure, // 20

fp\_sludge,

fa\_npk,

fa\_pk,

fa\_slurry,

fa\_ammoniumsulphate,

fa\_manure,

fa\_greenmanure,

fa\_sludge,

herbicide\_treat, // 29

growth\_regulator, // 30

fungicide\_treat,

insecticide\_treat, // 32

molluscicide,

row\_cultivation,

strigling, // 35

flammebehandling,

hilling\_up,

water,

swathing,

harvest, // 40

cattle\_out,

pigs\_out,

cut\_to\_hay,

cut\_to\_silage,

straw\_chopping, // 45

hay\_turning,

hay\_bailing,

stubble\_harrowing,

autumn\_or\_spring\_plough,

burn\_straw\_stubble, // 50

mow,

cut\_weeds,

strigling\_sow,

trial\_insecticidetreat,

trial\_toxiccontrol,

trial\_control,

syninsecticide\_treat,

cattle\_out\_low,

product\_treat,

glyphosate,

last\_treatment // Must be last, used for size computations

// of internal arrays etc.

} FarmToDo;


- CJT
- MSVC
- ALMaSS Working Source
- Landscape
- treatment.h
- Generated on Thu Jan 10 2013 13:15:35 for ALMaSS Skylark ODdox by
   1.8.1.1
